# Reported ultra-low lava viscosities from the 2021 La Palma eruption are potentially biased

**DOI:** 10.1038/s41467-023-42022-x

**Published:** 2023-10-16

**Authors:** Guillem Gisbert, Valentin R. Troll, James M. D. Day, Harri Geiger, Francisco J. Perez-Torrado, Meritxell Aulinas, Frances M. Deegan, Helena Albert, Juan Carlos Carracedo

**Affiliations:** 1 Department of Mineralogy, Petrology and Applied Geology, University of Barcelona, Barcelona, Spain; 2 Department of Earth Sciences, Natural Resources & Sustainable Development, Uppsala University, Uppsala, Sweden; 3 Instituto de Estudios Ambientales y Recursos Naturales, University of Las Palmas de Gran Canaria, Las Palmas de Gran Canaria, Spain; 4 Center of Natural Hazard and Disaster Science, Uppsala University, Uppsala, Sweden; 5 Scripps Institution of Oceanography, University of California San Diego, La Jolla, CA USA; 6 Institute of Earth and Environmental Sciences, University of Freiburg, Freiburg im Breisgau, Germany

**Keywords:** Volcanology, Natural hazards

**arising from** J. M. Castro & Y. Feisel. *Nature Communications* 10.1038/s41467-022-30905-4 (2022)

Magma viscosity is a major factor controlling volcanic eruptions and lava runout distances. An accurate characterisation of a given volcano or volcanic field is therefore fundamental for realistic forecasting of the impact of ongoing or future volcanic events. The 2021 eruption of Tajogaite volcano on La Palma (Canary Islands, Spain; 19/09/2021 to 13/12/2021) offers an exceptional volcanological test site due to the sustained visual, geophysical, and petrological monitoring that took place there (e.g., ref. ^1–5^). Access to the emitted materials throughout the eruption allowed detailed sampling of lava flows and proximal pyroclastic deposits, from which physical parameters of erupted lavas, such as viscosity, could be derived.

Magmas and lavas consist of three constituent phases: melt, solids and gas. Their effective viscosity is controlled by: (1) the viscosity of the melt, which in turn is controlled by its composition (e.g., melt viscosity increases with SiO_2_ and decreases with H_2_O contents) and temperature (viscosity decreases with increasing temperature); (2) percentage of entrained solids and their characteristics (e.g., size distribution, shape, density); (3) vesicle content and characteristics, including vesicle size distribution relative to the crystal population, and (4) the strain rate, which affects bubble behaviour^6–9^.

Ref. ^10^ presented estimates of the viscosity of effusive lavas of the 2021 Tajogaite eruption on La Palma based on experimental measurements and numerical modelling. Their estimates, with values of <10 to ~160 Pa·s, were claimed to represent the viscosity of lavas upon emission at the vent, and were used to suggest that these lavas had the lowest viscosity of all studied historical basaltic eruptions worldwide. However, after careful assessment, we argue that their lowest reported viscosities, on which the description of ultra-low viscosity for effusive lavas is based, result from sub-optimal methodology and input values during numerical modelling. As we discuss in detail below, the first and foremost cause for the unusually low presented viscosities is that the used melt temperatures (1150–1200 °C) and water contents (>0.5 wt.%) correspond to equilibrium conditions in a deep magma reservoir and not to surface or near-surface vent conditions. Consequently, reported viscosities do not represent those of lavas upon emission at the vent as claimed, but rather magma viscosities at depth, prior to final ascent and emission, which are considerably lower due to higher melt temperature and water contents. Moreover, the viscosity estimates presented by ref. ^10^ are affected by additional factors also leading to lower viscosities, including the use of the chemical composition of bulk tephra instead of glass (=melt), and of crystal contents derived from pyroclasts instead of from contemporary lava flows, as input parameters for numerical modelling.

To prevent the use of inaccurate viscosity values in lava flow propagation modelling for hazard assessment on La Palma and elsewhere in the future it is important to review the results of ref. ^10^, and to critically assess the most likely viscosities of lavas upon emission at the vent. Therefore, we discuss the method and input values chosen by these authors, and subsequently use more appropriate input values derived from observations on the eruption and lava samples to calculate a revised viscosity range for Tajogaite lavas.

## Temperature

Using mineral-melt geothermobarometry, ref. ^10^ derived temperatures of 1150–1160 °C (crystal rims) to 1190 °C (crystal cores) at 7–10 kbar (ca. 24–34 km depth) for the tephra-forming magmas studied in their work. These results are used by the authors to suggest a potential eruptive temperature of the tephra of 1150–1200 °C, with the additional suggestion that the eruptive temperatures of effusive lavas were higher than for contemporary pyroclasts based on the visual observation of higher radiance in the lavas.

Regarding the suggested higher eruptive temperatures of magmas feeding lava flows compared to those feeding pyroclastic activity, visual observation of radiance is not likely to be accurate because, although the effusive lava vents could be directly observed, those of ejected pyroclasts could not due to being located within craters. We note instead that drone footage of vents (e.g., https://volcan.lapalma.es/pages/multimedia) shows equivalent radiances for tephra and lava.

**Fig. 1:**
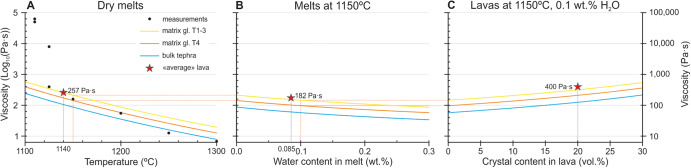
Melt and lava viscosities calculated using bulk tephra and glass compositions from ref. ^10^ and the model of ref. ^7^. **A** Viscosity of anhydrous melts at temperatures from 1100 to 1300 °C. Experimentally-derived viscosities reported in ref. ^10^ are provided for reference depicted with the “measurements” symbol. **B** Viscosity of melts at 1150 °C with water contents from 0 to 0.3 wt.%. **C** Effective viscosity of lavas consisting of melts with 0.1 wt.% H_2_O content at 1150 °C, and crystals with abundances from 0 to 30 vol.%. The <<average>> lava viscosity is calculated using likely average values for the studied parameters: glass T1-3 melt composition, 1140 °C, 0.085 wt.% H_2_O content in melt, 20 vol.% crystal content.

In terms of the potential eruptive temperatures, the temperatures derived from geothermobarometry by ref. ^10^ represent equilibrium conditions in the magma reservoir at depth (>20 kilometres below the surface, see above) and not the temperatures upon lava emission at the vents. If crystal-derived temperatures are to be employed to estimate eruptive temperatures, only those derived from the outermost crystal rims ought to be chosen because they indicate the last equilibration temperature at depth prior to final ascent and eruption. In addition, some subsequent cooling is to be expected during the final ascent due to adiabatic decompression and conductive heat loss even in a warmed plumbing system two months into the eruption. As a reference, maximum temperatures of 1140 °C were measured for lava flows by the PEVOLCA during the eruption^1,11,12^. These are very similar to temperatures derived from crystal rims by ref. ^10^ as well as to lava temperatures determined during the hottest phases of the 2018 Kilauea flank eruption (~1140–1145 °C)^13,14^, the 1984 Mauna Loa eruption (1140 ± 3 °C)^15^, or the Piton de la Fournaise July–August 2015 eruption (1146 °C at the vent)^16^. Therefore, lava temperatures at the vent during the Tajogaite eruption were more likely below 1150 °C, and so markedly lower than those employed by ref. ^10^ for viscosity calculations (1150–1200 °C). These temperature differences have a significant impact on lava viscosity determinations (see Fig. 1A for reference on the impact of temperature on melt viscosity).

## Water content of melts

**Fig. 2:**
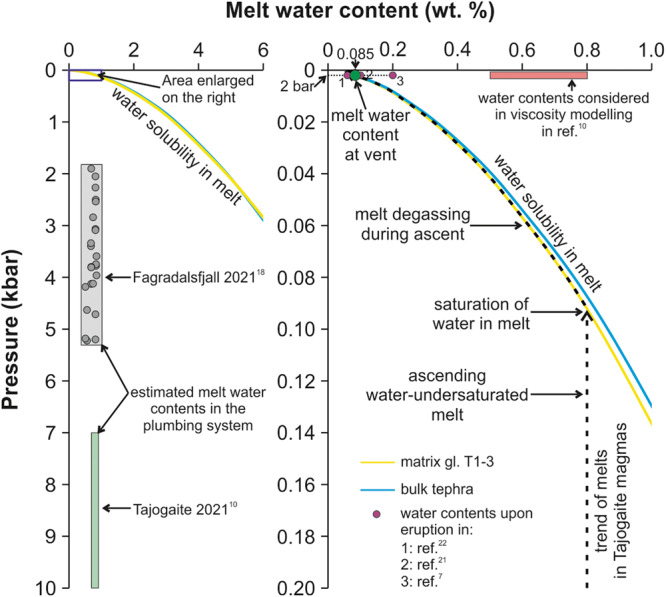
Water contents and solubility in basaltic and basanitic melts. Water contents in the Tajogaite 2021 eruption melts are those reported by ref. ^10^. Water contents in melts from the Fagradalsfjall 2021 eruption reported by ref. ^18^ are provided for reference. Solubility curves are calculated for melt compositions equivalent to the composition of bulk tephra and glass in sample T1-3 in ref. ^10^ using the model by ref. ^19^ at 1150 °C. The diagram on the right shows an enlargement of the area marked with a blue square on the left. The melt water content employed by ref. ^10^ in numerical modelling of melt viscosity is represented by a red rectangle; note that it is above the solubility curve and thus unrealistic for surface or near-surface settings.

Using plagioclase-melt hygrometry, a melt water content of ~0.8 wt.% H_2_O at 7–10 kbar was calculated by ref. ^10^, based on which a potential melt water content of 0.5–0.8 wt.% H_2_O was used for numerical modelling of viscosity at the vent upon emission. Again, these contents represent equilibrium conditions in a deep magma reservoir, not at the surface or in a near-surface vent setting. Because water solubility in a melt is strongly dependent on pressure and decreases dramatically in near-surface environments, the melt water content at the vent must have been significantly lower than 0.8% (cf. ref. ^17,18^) (see Fig. 2 for reference).

While direct measurement of the water content in melts during eruption is currently impossible, numerical modelling using quenched glass compositions provides reasonable approximations. Water solubility increases with the silica content of a melt^19^, so rhyolitic melts can be used as a reference of the high end of melt water contents in lavas at the vent. A rhyolitic melt can typically hold up to ~0.2 wt.% H_2_O upon eruption (Fig. 10 in ref. ^7^; estimation using MELTS). Because at Tajogaite volcano the melt was basanitic^10,20^, its water content must have been considerably lower. Using the model by ref. ^19^ at 1150 °C and 2 bars (vent conditions with only a few metres depth of lava), and considering a water-only vapour phase, the water content of a basanite melt with the bulk tephra and glass compositions of ref. ^10^ would be ~ 0.085 wt.% H_2_O (Fig. 2). This is nearly an order of magnitude lower than the 0.5–0.8 wt.% H_2_O used by ref. ^10^ (see comparison in Fig. 2). Our estimated H_2_O content is consistent with measured or estimated H_2_O contents in basaltic/basanitic melts in lavas at other locations, such as 0.1 wt.% H_2_O in the 1984 Mauna Loa eruption^21^ and 0.06 wt.% H_2_O for a lava flow of the Western Volcanic Zone, Iceland^22^. Thus, the water content of melt in lavas at the vent during the Tajogaite eruption was likely much lower than the used in ref. ^10^ which, as with temperature, has a strong impact in melt viscosity determination (cf. ref. ^6,7^; see Fig. 1B for reference on the impact of water content on melt viscosity).

## Opportunities and challenges related to the use of tephra for experimental and numerical determination of effusive lava viscosity

Ref. ^10^ studied ash and lapilli samples collected at El Paso (3–4 km from the vent) and Santa Cruz de La Palma (12–13 km distant) that were deposited between 14 and 20/11/2021. Because fine pyroclasts solidify rapidly during transport in the atmosphere, they are likely to record the texture, mineralogy, and melt chemistry of the magma at the moment of emission better than lava flow samples, which undergo slower cooling during and after emplacement. However, several implications need to be discussed when using tephra as study material to estimate the viscosity of contemporaneous lava flows as was the case in ref. ^10^.

### Experimental measurements

For the experimental determination of lava viscosities by cooling a melt from temperatures above the liquidus, the starting materials should have identical chemical composition to the lava that is being studied. When working with lava flow samples this is usually straightforward, but when using bulk tephra samples to approximate lava it should be noted that the chemical composition of a tephra need not be representative of that of the originally erupted magma.

For instance, in a volcanic plume pyroclasts fractionate according to their settling velocities, which are primarily controlled by their size, density, and shape (e.g., ref. ^23^). Because crystals are typically denser than glass, pyroclasts with higher crystal contents will tend to settle in a higher proportion from the eruption plume during transport, producing a pyroclast fractionation that results in an overall enrichment of crystals in proximal tephra and in glass in more distal ones (Supplementary materials). Consequently, bulk tephra chemical composition tends to become more evolved (i.e., silica-rich) with distance from the vent, progressively approaching pure glass compositions (cf. ref. ^24^). Consistently, at La Palma, Tajogaite bulk tephras have been shown to have higher SiO_2_ contents than contemporary lavas^20^. The use of tephras for experimental viscosity measurement will therefore result in distorted values and, depending on sampling location (proximal versus distal), will either lead to lower or higher viscosity estimates relative to the original magma. In this sense, the use of bulk tephra as in ref. ^10^ is not optimal, and, instead, lava flow samples ought to be prioritised. In the case of La Palma, the compositional differences between tephra and lava flow samples are relatively small, which results in a smaller effect on viscosity determinations than other factors discussed here; as a reference, see in Fig. 1 the changes in viscosity related to composition—bulk tephra versus glass—compared to those resulting from the overestimates on temperature and water content.

### Numerical modelling

To estimate the effective viscosity of an effusive lava through numerical modelling, the viscosity of the melt has to be established first. Then, the effect of solids (e.g., crystals, xenoliths) and vesicles needs to be added (e.g., ref. ^6–9,25^).

#### Melt composition

The lowest viscosities reported in ref. ^10^ are estimated using bulk tephra data as melt composition. However, because erupted magmas typically show some degree of crystallinity, the melt in them presents a composition which is usually more evolved (i.e., richer in silica) than that of the bulk magma. As a result, melt viscosities estimated from bulk tephra compositions (or bulk lava if working with lava flow samples) will be lower than those obtained if the composition of the actual melt is used.

Obtaining the composition of the melt component in effusive lava upon emission is not straightforward due to further lava crystallisation during flow and final cooling before sampling may become possible. Conversely, pyroclasts usually solidify upon emission, which means that the chemical composition of the glass in the tephra typically is more representative of that of the melt at the time of eruption. In this sense, pyroclasts become a valuable source of information about syn-eruptive melt compositions.

However, the composition of glass in tephra represents the melt feeding explosive eruptive activity, not that in contemporary effusive lava, which may differ significantly. In Tajogaite volcano several vents were usually simultaneously active displaying variable behaviour (effusive and explosive)^1,11,26^ (Fig. S.1 in Supplementary materials). This indicates a heterogeneous distribution of volatiles within the plumbing system, with gas-rich magmas feeding explosive vents, and more degassed magmas feeding effusive lavas. In effusive lavas, lower volatile content (resulting in increased liquidus temperatures) coupled with likely lower ascent velocities (to allow for degassing; resulting in increased time available for cooling and crystallisation), should cause higher crystallinity and consequently a more evolved (i.e., richer in Si) melt composition (e.g., ref. ^27,28^) (see the difference in glass relative to bulk tephra composition in ref. ^10^). Thus, melt viscosity estimates derived from glass in tephra can only be used as a proxy for that of contemporary effusive lavas, and in any case should be regarded as a minimum.

#### Crystal content

**Fig. 3:**
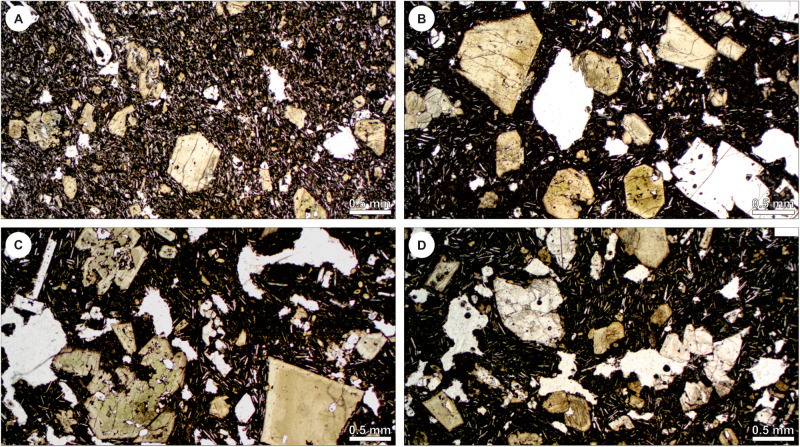
Photomicrographs of lava flow samples that erupted contemporaneously to tephras studied in ref. ^10^. **A** Sample LP-21-75, erupted on November 10th. **B**, **C** Sample LP-21-77, erupted on November 15th. **D** LP-21-81, erupted on November 15th. Chemical analyses for these samples are provided in ref. ^3^.

Ref. ^10^. reports a 6–16 vol.% (normalised to vesicle-free) crystal content in the studied tephras, which is used as a proxy for that in effusive lavas at the vent. However, as argued above, the crystal content in contemporary lavas could differ significantly. Therefore, we established the modal content of crystals in four different lava flow samples (Fig. 3) contemporaneous (10/11/21 to 01/12/21) to the tephras investigated in ref. ^10^. The results are shown in Table 1. As evidenced by SEM images of tephras in ref. ^10^, the crystal content of magma at the vent included the phenocrysts and microphenocrysts, plus a portion of the plagioclase microlites and pyroxene and olivine microcrystals. Taking this into account, and based on our crystal content estimations, we consider 15 vol.% as a minimum crystal content for effusive lavas at La Palma, with a more common content ~≥20 vol.%. The effect of higher crystal contents in effusive lavas is twofold: (1) higher melt viscosity due to more evolved melt composition; and (2) higher effective lava viscosity due to more solids in suspension (cf. ref. ^7,29,30^); and thus results in higher effective viscosity even at equivalent bulk chemical composition.

**Table 1:** Modal contents of phenocrysts, groundmass and vesicles in three lava flow samples emitted on November 10th to December 1st; obtained by point counting (*n* > 1000) of thin sections

Sample	LP-21-75	LP-21-77	LP-21-81	LP-21-82
**Emission date**	10/11/2022	15/11/2022	15/11/2022	1/12/2022
**Modal contents (vol.%)**				
Phenocrysts + microphenocrysts (ol+px+oxides; >100 μm)	12.7	12.8	10.7	9.9
Microcrystals (ol+px; 10–100 μm)	10.8	5.1	5.0	4.8
Microlites (pl)	14.1	12.2	8.4	6.3
Fine groundmass (<10 μm)	54.9	58.6	67.7	62.4
Vesicles	7.4	11.4	8.2	16.6
**Vesicle-free modal contents (vol.%)**				
Phenocrysts + microphenocrysts (ol+px+oxides; >100 μm	13.7	14.4	11.6	11.9
Microcrystals (ol+px; 10–100 μm)	11.7	5.7	5.4	5.7
Microlites (pl)	15.3	13.7	9.2	7.6
Fine groundmass (<10 μm)	59.4	66.2	73.8	74.8

## Recalculating viscosities

We have re-evaluated the viscosity of effusive lavas emitted during the 2021 Tajogaite eruption by employing the same numerical model as ref. ^10^—that is, ref. ^7^ (GRD)—, but using as input what we consider more appropriate temperature, volatile/water content, melt composition, and crystal content values (Table 2, Fig. 1). The effect of gas bubbles on the effective viscosity of lava has, however, not been considered in this re-evaluation, as this was also not considered by ref. ^10^. Gas bubbles play an important role in controlling lava viscosity, although the assessment of their effect in three-phase mixtures (melt + crystals + bubbles) is complex and not yet well constrained. This is because of the deformable and compressible character of bubbles, the effect of vesicle size distribution relative to the crystal population, and the control of strain rate on bubble behaviour^6,8,9^.

**Table 2:** Melt and lava viscosities calculated using bulk tephra and glass compositions from ref. ^10^, the model by ref. ^7^ for melt viscosity, and that of ref. ^31^ for lava effective viscosity (melt + crystals)

**Dry melt viscosity (Pa·s) at different temperatures**									
Temperature (°C)	1300	1275	1250	1225	1200	1175	1150	1125	1100
Bulk tephra	8	11	16	23	35	55	87	143	242
Glass in T4	13	18	26	38	58	89	142	233	395
Glass in T1-3	19	27	40	58	87	135	212	344	576
**Melt viscosity (Pa·s) at 1150 °C and different water contents**									
Water content (wt.%)	0	0.05	0.10	0.15	0.20	0.25	0.30	0.50	0.80
Bulk tephra	87	69	57	49	43	38	34	24	17
Glass in T4	142	115	97	83	72	64	57	40	28
Glass in T1-3	212	173	145	125	109	96	86	60	40
**Lava viscosity (Pa·s) at 1150 °C, with 0.1 wt.% water in melt, and different crystal contents**									
Crystal content (vol.%)	0	5	10	15	20	25	30		
Bulk tephra	57	68	82	100	125	161	215		
Glass in T4	97	114	137	168	210	271	362		
Glass in T1-3	145	172	207	253	317	409	546		

Regarding input melt composition, the glass compositions provided by ref. ^10^, and not that of the bulk tephra, were used in our recalculation. Although the GRD model is calibrated for melts with <3 wt.% TiO_2_ and tephras in ref. ^10^ show 3.69 (bulk) to 4.27 (glass in Tephra 4) wt.% TiO_2_, the agreement between numerical and experimental values at high temperatures (>1250 °C, 100% melt) in ref. ^10^ indicates that it can be relied upon for Tajogaite melts.

Our results show that at 1150 °C (high end of the likely eruption temperatures) calculated dry melt viscosities are 142 (glass in sample T4) and 212 (glass in sample T1-3) Pa·s (Table 2). Melt viscosities calculated using glass compositions are 1.6 to 2.5 times higher than those estimated from bulk tephra (87 Pa·s), highlighting the importance of using glass rather than bulk tephra/lava compositions in numerical modelling. At this temperature, water in the melt reduces viscosity by a factor of 1.5 at 0.1 wt.% H_2_O and by a factor 2 at 0.2 wt.%. Considering a 0.1 wt.% H_2_O water content (slightly above calculated contents for a conservative safety margin accounting for incomplete equilibration and/or higher lava thickness), viscosities of 97 (glass in sample T4) and 145 (glass in sample T1-3) Pa·s are obtained. For comparison, in Table 2 we also list the strongly reduced viscosities obtained if water contents of 0.5–0.8 wt.% H_2_O are employed. If the effect of crystal content on the effective viscosity for the melt at 1150 °C and 0.1 wt.% H_2_O is then calculated—using the model by ref. ^31^ and a maximum packing fraction of 0.62 following ref. ^10^—, estimated lava viscosities increase by a factor of 1.7 at 15 vol.% crystals and 3.8 at 30 vol.%. Considering 15 vol.% crystals as a minimum value for the effusive lavas at the vent, this results in effective lava viscosities of 168 (derived from glass in sample T4) and 253 (derived from glass in sample T1-3) Pa·s (Table 1).

These calculated viscosities are over an order of magnitude higher than the lowest suggested values in ref. ^10^ (down to <10 Pa·s), which are used by the authors to characterise the Tajogaite basanite lavas as ultra-low viscosity and which result in abnormally high estimated Reynolds numbers. Even if highly unlikely extreme input values were used—glass in sample T4 (less evolved glass), 1160 °C (maximum T derived from rim crystal-melt thermometry), 0.1 wt.% H_2_O in melt, and 10 vol.% crystals (middle of the crystallinity range reported in ref. ^10^, significantly below our observed lava crystallinities)—the obtained effective viscosity would be 115 Pa·s, still an order of magnitude higher than 10 Pa·s.

However, our estimated viscosities (168–253 Pa·s) are close to those experimentally measured by ref. ^10^ at 1150 °C (158 Pa·s) (even though the crystallising assemblage in their experiments is different to that in the natural rocks, and despite the factors discussed in “Opportunities and challenges related to the use of tephra for experimental and numerical determination of effusive lava viscosity”). Moreover, they are within the range of common basaltic ocean island lavas, such as those on Hawaii (e.g., ref. ^32,33^ and references therein). Therefore, we consider our new lava viscosity estimates to be more realistic, although we note that they likely represent only a minimum for effusive lavas at the vent during the Tajogaite eruption because they have been calculated using glass-in-tephra compositions. In this regard, viscosities derived from glass in sample T1-3 likely represent the best approximation.

To provide a reference of what we consider the most likely characteristics of effusive lavas at the vent during the Tajogaite eruption, we estimate the viscosity of a lava with a melt based on glass in sample T1-3, at 1140 °C, 0.085 wt.% H_2_O in the melt, and 20 vol.% crystal content. This calculation results in a lava effective viscosity of 400 Pa·s (star in Fig. 1), which coincidentally overlaps the range of viscosities estimated for the 2018 flank eruption of Kilauea (250–1150 Pa·s)^14^.

After emission at the vent, lava viscosity likely increased rapidly during downslope flow. The global study of lava flow fields has shown that, during surface flowage, melt viscosity increases due to degassing (=lower melt water contents) and degassing- and cooling-induced crystallisation (=more evolved melt compositions). Additionally, higher crystallinity further increases effective lava viscosity and yield strength (e.g., ref. ^22,34^). Evolution of vesicularity and bubble shape and size by degassing during flow may further modify (decrease or increase) the effective viscosity of lava depending on whether bubbles are deformed or not^16,35^, but this is an effect not considered here, nor in ref. ^10^. Relevant for La Palma, the experiments in ref. ^10^ provide evidence for the potential effect of thermal cooling during flow on the viscosity of Tajogaite lavas. In them, artificial lavas show a marked viscosity increase below 1125 °C due to increased crystallisation. Because this temperature is only ca. 25 °C below the likely eruptive temperatures, lava viscosity may have significantly increased soon after emission from the vent sites. Our estimates are therefore consistent with the formation of predominant a’a’ lava flows throughout the lava flow field during the eruption^1^ (Fig. S.1). Additionally, they do not preclude the presence of standing waves in lava channels, because standing waves can also be caused by flow at high speeds over steep slopes (e.g., near the volcanic cone) and thus do not necessitate ultra-low viscosities (cf. ref. ^33^).

We therefore conclude that: (1) the viscosity estimates presented by ref. ^10^ should be considered a first approximation to potential magma viscosities within the deep (>20 km) plumbing system of Tajogaite volcano, which represents valuable information towards the study and understanding of this volcano, but cannot serve as an estimate of effusive lavas at the vent; (2) the viscosity of Tajogaite lavas on La Palma in 2021 was not in any way unusual compared to other oceanic island basaltic eruptions, neither at the vent nor in the flow field facies, which needs to be considered in future numerical modelling of eruptive behaviour on La Palma and other volcanoes with similar magma composition.

Finally, we would like to stress the importance of choosing the right materials (e.g., glass instead of bulk tephra for melt composition, lava flow samples for crystallinity estimation and experimental viscosity measurement) and temperature and water values (near-surface instead of reservoir conditions) when assessing the viscosity of magmas that are feeding lava flows. This will allow accurate viscosity values to be obtained and help to produce the most realistic modelling of potential lava flow propagation, which is key to a robust and accurate hazard assessment.

### Supplementary information

Supplementary Information
