# Supplementary material for: Reported ultra-low lava viscosities from the 2021 La Palma eruption are potentially biased
Source: Nat Commun. 2023 Oct 16;14:6453. doi: 10.1038/s41467-023-42022-x (PMC10579349; doi:10.1038/s41467-023-42022-x)
Supplement: Supplementary file 1 — Supplementary Information [file 41467_2023_42022_MOESM1_ESM.pdf]

# REPORTED ULTRALOW LAVA VISCOSITIES FROM THE 2021 LA PALMA ERUPTION ARE POTENTIALLY BIASSED

Guillem Gisbert[1], Valentin R. Troll[2,3,4], James M.D. Day[5], Harri Geiger[6], Francisco J. Perez-Torrado[3], Meritxell Aulinas[1], Frances M. Deegan[2,4], Helena Albert[1], Juan Carlos Carracedo[3]

[1]Department of Mineralogy, Petrology and Applied Geology, University of Barcelona, Barcelona, Spain

[2]Department of Earth Sciences, Natural Resources & Sustainable Development, Uppsala University, Uppsala, Sweden

[3]Instituto de Estudios Ambientales y Recursos Naturales, University of Las Palmas de Gran Canaria, Las Palmas de Gran Canaria, Spain

[4]Center of Natural Hazard and Disaster Science, Uppsala University, Uppsala, Sweden

[5]Scripps Institution of Oceanography, University of California San Diego, La Jolla, USA

[6]Institute of Earth and Environmental Sciences, University of Freiburg, Freiburg im Breisgau, Germany

## SUPPLEMENTARY MATERIALS

### Pyroclast fractionation during transport

In a volcanic plume, pyroclasts fractionate according to their settling velocities, which are primarily controlled by their size, density, and shape (e.g., ref.[1]). Because crystals and glass in pyroclasts have different densities, for a given magma composition (chemical and mineralogical) and pyroclast vesicularity, the density of individual pyroclasts is controlled by their relative crystal and glass contents. For instance, crystals in basaltic rocks are denser than coexisting glass (e.g., olivine, 3.27 g/cm$^3$; diopside 3.40 g/cm$^3$; magnetite 5.15 g/cm$^3$; anorthite 2.73 g/cm$^3$; which compare to basaltic glass, 2.66-2.85 g/cm$^3$; ref.[2-5]). Consequently, pyroclasts with higher crystal contents will be denser and tend to settle in a higher proportion from the eruption plume during transport, which will result in an overall enrichment in the glass content of the remaining ash and lapilli in the eruption plume. This density-controlled fractionation is also observed in more evolved magma compositions (e.g., ref.[6]) and increases with distance travelled. The preferential settling of crystal-rich pyroclasts usually results in proximal (s.l.) tephras with crystal contents higher than in the original magma. In contrast, tephras in more distal (s.l.) locations show lower crystal contents. As a result of this process, bulk tephra chemical composition tends to become more evolved (i.e. silica-rich) with distance from the vent, progressively approaching glass compositions (cf. ref.[6]).

**Supplementary figure**

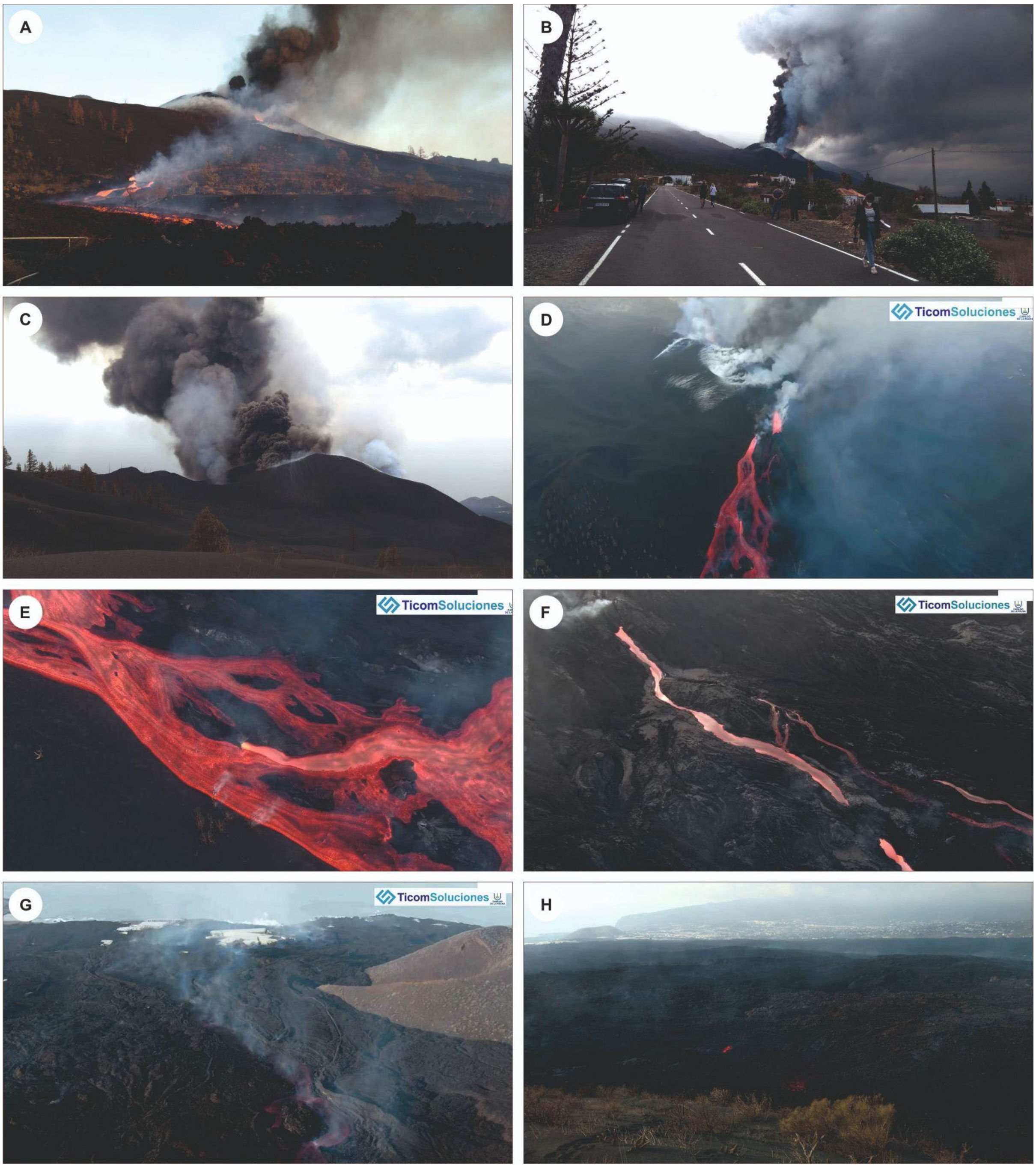


**Figure S.1**. Images showing representative volcanic activity during the 2021 Tajogaite eruption. A) Northern flank of the volcano on October 2nd; note the Strombolian activity on the cone, and the simultaneous emission of effusive lavas from two vents at lower altitude. B) Northern flank on October 27; dark eruptive column above a vent with Strombolian activity, and a white gas-rich column above a lava fountain feeding lava flows. C) Western flank on October 27; from left to right: grey gas plume over a vent emitting only gas, dark eruptive column above a vent with Strombolian activity, and white gas above a vent with lava fountaining feeding lava flows. D) Drone footage from October 25 showing the alignment of vents on the volcano with simultaneous explosive and effusive activity. E) Drone footage from November 11 showing the effusive emission of lavas and their fast transition to a’a’ type lava flows. F) Drone footage from November 11; note that pahoehoe-like features are restricted to

proximal overflows by the lava channels close to the vent, with the rest of the lava field dominated by a'a' lavas. G) Drone footage of the lava field from November 11; note the complete predominance of a'a' lava flows. H) Picture of the lava field from Montaña Cogote on November 1st showing the a'a' character of the field, including an active lava flow channel. Ground pictures: own. Drone footage: Ticom Soluciones for the La Palma local authorities, publicly available at https://volcan.lapalma.es/pages/multimedia.

**References**

1. Folch A, Costa A, Macedonio G. FALL3D: A computational model for transport and deposition of volcanic ash. *Computers & Geosciences* **35**, 1334-1342 (2009).
2. webmineral.org
3. Kuryaeva RG, Kirkinskii VA. Influence of high pressure on the refractive index and density of tholeiite basalt glass. *Physics and Chemistry of Minerals* **25**, 48-54 (1997).
4. Clark AN, Lesher CE, Jacobsen SD, Wang Y. Anomalous density and elastic properties of basalt at high pressure: Reevaluating of the effect of melt fraction on seismic velocity in the Earth's crust and upper mantle. *Journal of Geophysical Research: Solid Earth* **121**, 4232-4248 (2016).
5. Liu J, Chang Z, Wang L, Xu J, Kuang R, Wu Z. Exploration of basalt glasses as high-temperature sensible heat storage materials. *ACS Omega* **5**, 19236-19246 (2020).
6. Lerbekmo JF, Campbell FA. Distribution, composition, and source of the White River Ash, Yukon Territory. *Canadian Journal of Earth Sciences* **6**, 109-116 (1969).
